# Inhibition of the mammalian target of rapamycin (mTOR) in advanced pancreatic cancer: results of two phase II studies

**DOI:** 10.1186/1471-2407-10-368

**Published:** 2010-07-14

**Authors:** Milind M Javle, Rachna T Shroff, Henry Xiong, Gauri A Varadhachary, David Fogelman, Shrikanth A Reddy, Darren Davis, Yujian Zhang, Robert A Wolff, James L Abbruzzese

**Affiliations:** 1 Department of Gastrointestinal Medical Oncology, UT-M D Anderson Cancer Center, Houston, TX, USA; 2 Center for Cancer and Blood Disorders, Dallas, TX, USA; 3 Apocell Inc, 2575 West Bellfort, Suite 190, Houston, TX, USA

## Abstract

**Background:**

The phosphoinositide 3-kinase (PI3K)/Akt pathway is constitutively activated in pancreatic cancer and the mammalian target of rapamycin (mTOR) kinase is an important mediator for its signaling. Our recent *in vitro *studies suggest that prolonged exposure of pancreatic cancer cells to mTOR inhibitors can promote insulin receptor substrate-PI3K interactions and paradoxically increase Akt phosphorylation and cyclin D1 expression in pancreatic cancer cells (negative feedback loop). The addition of erlotinib to rapamycin can down-regulate rapamycin-stimulated Akt and results in synergistic antitumor activity with erlotinib in preclinical tumor models.

**Methods:**

Two studies prospectively enrolled adult patients with advanced pancreatic cancer, Eastern Cooperative Oncology Group performance status 0-1, adequate hematologic, hepatic and renal parameters and measurable disease. In Study A, temsirolimus was administered intravenously at 25 mg weekly. In Study B, everolimus was administered orally at 30 mg weekly and erlotinib was administered at 150 mg daily. The primary endpoint in both studies was overall survival at 6 months. Secondary endpoints included time to progression, progression-free survival, overall survival, response rate, safety and toxicity. Pretreatment tumor biopsies were analyzed by immunofluorescence and laser scanning cytometry for the expression of pmTOR/mTOR, pAkt/Akt, pErk/Erk, pS6, p4EBP-1 and PTEN.

**Results:**

Five patients enrolled in Study A; Two patients died within a month (rapid disease progression and hemorrhagic stroke, respectively). One patient developed dehydration and another developed asthenia. Sixteen patients enrolled in Study B.: 12 males, all ECOG PS = 1. Median cycles = 1 (range 1-2). Grade 4 toxicity: hyponatremia (n = 1), Grade 3: diarrhea (n = 1), cholangitis (n = 3), hyperglycemia (n = 1), fatigue (n = 1). Grade 2: pneumonia (n = 2), dehydration (n = 2), nausea (n = 2), neutropenia (n = 1), mucositis (n = 2) & rash (n = 2). Four patients were hospitalized. Progressive disease occurred in 15 and 1 was non-evaluable. Pretreatment biopsies revealed a higher pAkt/Akt ratio in tumor specimens that in nonmalignant pancreatic tissue. No such trends were noted for the other biomarkers.

**Conclusions:**

Neither study with mTOR inhibitors demonstrated objective responses or disease stability. The negative feedback loop resulting from mTOR inhibition may account for the disease progression and toxicity noted in these studies. Future strategies should aim for a broader targeting of the PI3K pathway in pancreatic cancer.

**Trial Registration:**

**Trial registration: Study A**: NCT 0075647. **Study B**: NCT00640978

## Background

Gemcitabine, the standard frontline chemotherapeutic agent for advanced pancreatic cancer, was approved by the Food and Drug Administration (FDA) over a decade ago. Gemcitabine confers marginal survival benefit, although one randomized trial reported 'clinical benefit response' in 24% of patients with advanced pancreatic cancer [1]. No 'standard' second-line options for treating this disease have been adopted, although 5-fluorouracil, capecitabine, or a capecitabine + oxaliplatin combination is commonly used [2]. Based on our knowledge of pancreatic carcinogenesis, molecular targeting may lead to therapeutic gains in this disease. The epidermal growth factor receptor (EGFR) and its downstream signaling intermediates, the mitogen-activated protein kinase kinase (MEK), extracellular signal-regulated kinase (Erk) and phosphoinositide 3-kinase (PI3K)/Akt signaling pathways, play important roles in cellular proliferation, survival (inhibition of apoptosis) and drug resistance in pancreatic cancer. We and others have demonstrated that the PI3K/Akt pathway is constitutively activated in pancreatic cancer, thereby activating two important transcription factors, nuclear factor-kappa beta and c-myc [3].

Although the precise mechanism is unclear, the mammalian target of rapamycin (mTOR), a protein kinase, is the principal mediator of signals arising from PI3K/Akt-driven mitogen stimulation [4]. Activation of mTOR involves Akt and the tuberous sclerosis complex. Mutations in these components or in the phosphatase and tensin homolog (PTEN), a tumor suppressor and negative regulator of PI3K, may result in their dysregulation and thus contribute to the pathophysiology of cancer [5]. The mTOR pathway is also involved in the production of pro-angiogenic factors, including vascular endothelial growth factor (VEGF), that enhance endothelial cell growth and proliferation. Through the activation of its downstream mediators including the 40S ribosomal S6 kinases, mTOR can also activate hypoxia-inducible factor 1 (HIF-1). Inhibition of mTOR is therefore being explored as an anti-cancer strategy for several types of human malignancies, including pancreatic cancer.

Inhibition of EGFR by its oral tyrosine kinase inhibitor, erlotinib, has also been shown to have a therapeutic effect on pancreatic cancer. The results of a recent phase III clinical trial suggested that erlotinib in combination with gemcitabine was associated with a significant overall survival improvement over single-agent gemcitabine [6]. The sensitivity of cancer cell lines to erlotinib may depend on the inhibition of the PI3K/Akt pathway. Buck et al. investigated whether rapamycin, an mTOR inhibitor, could enhance the sensitivity of non-small-cell lung, pancreatic, colon and breast cancer cell lines to erlotinib [7]. Erlotinib inhibited Erk, Akt and S6 kinase in only the most sensitive cancer cell lines. Rapamycin could fully inhibit S6 kinase in all cell lines but simultaneously activated Akt. However, the rapamycin/erlotinib combination was able to down-modulate rapamycin-stimulated Akt activity. The rapamycin-erlotinib combination resulted in synergistic cancer cell growth inhibition *in vitro *and *in vivo*.

We investigated the role of mTOR inhibition and combined mTOR-EGFR inhibition in pancreatic cancer in the following two prospective clinical trials: Trial A, a phase II study of the mTOR-inhibitor, temsirolimus (CCI-779), and Trial B, a phase II study of the mTOR-inhibitor, everolimus (RAD001) + erlotinib combination. The dose of temsirolimus was based on its currently approved dose for the treatment of renal cancer. The dose of everolimus + erlotinib combination was based on a phase I study conducted at our institution [8].

## Methods

### Study populations

Eligibility criteria for both studies: adult [Au: >18 years; metastatic pancreatic adenocarcinoma; Eastern Cooperative Oncology Group (ECOG) performance status 0 or 1; measurable disease; adequate hematologic, hepatic and renal function (leukocytes ≥3,000/μl, absolute neutrophil count ≥1,500/μl, platelets ≥100,000/μl, hemoglobin ≥9 g/dL, total bilirubin ≤1.5 mg/dl, aspartate aminotransferase (AST) ≤230 IU/L and alanine aminotransferase (ALT) ≤280 IU/L for subjects with documented liver metastases; AST ≤115 IU/L and ALT ≤140 IU/L for subjects without evidence of liver metastases; and creatinine ≤1.5 mg/dl in men and ≤ 1.2 mg/dl in women). Patients in study B were required to have received at least one prior gemcitabine-based regimen. Men and premenopausal women were advised to use adequate contraception. Prior chemotherapy or radiation must have been completed at least two weeks before enrollment.

Excluded from the studies were patients receiving steroids or immunosuppressive medications and those with hyperlipidemia (cholesterol ≥300 mg/dL and triglycerides ≥ 2.5 × ULN); uncontrolled brain metastases; other malignancies within the past 3 years, except for adequately treated cervical carcinoma or basal or squamous cell skin carcinomas; and uncontrolled medical conditions, including cardiorespiratory illness, infectious diseases and bleeding diathesis. Pregnant or nursing women were also excluded. Immunization with attenuated live vaccines within one week of study entry or during the study period was not permitted. Institutional Review Board approval was obtained for both the studies. All patients signed an informed consent document.

### Treatment

Study A: Patients received 25 mg of temsirolimus once weekly, infused intravenously over 30 minutes on days 1, 8, 15 and 22. Diphenydramine was also administered at a dose of 25 mg intravenously 30 minutes prior to infusion.

Study B: Patients received 30 mg of everolimus 30 mg orally once weekly in combination with 150 mg of erlotinib daily. The treatment cycle lasted 28 days for both studies. Treatment was continued until disease progression or unacceptable toxicity. Staging radiological studies were performed after every 2 cycles. Both were open-label, single-arm phase II studies conducted at The University of Texas M. D. Anderson Cancer Center. Treatment was administered on an outpatient basis. The primary outcome measure was overall survival (OS) at 6 months. Secondary outcome measures included time to progression (TTP), progression-free survival (PFS), response rate, safety and toxicity. Response was assessed by the Response Evaluation Criteria in Solid Tumors (RECIST) [9].

#### Dosage Adjustments

Study A: In instances of grade 2 or higher hematologic toxicity or grade 3 or higher non-hematologic toxicity (as per National Cancer Institute Common Toxicity Criteria, version 3.0) related to the study agent, treatment was withheld until toxicity recovery and then re-initiated with a 5 mg/week dose reduction; the minimum dose allowed was 15 mg/week.

Study B: In instances of clinically significant grade 2 or higher non-hematological toxicities or grade 3 or 4 hematological toxicities, everolimus was interrupted until toxicity recovery and re-introduced with a 10 mg dose reduction. Treatment was discontinued in patients with recurrent grade 3 or 4 toxicities or treatment interruption >2 weeks. Doses of erlotinib were reduced in 50-mg decrements; the minimum dose allowed was 50 mg. In patients with grade 3 or 4 diarrhea, erlotinib was interrupted until resolution and re-introduced with a 50 mg dose reduction. Rash was treated with topical and/or systemic antibiotics. In those with persistent grade 3 rash, dosage of erlotinib was reduced as above. Study treatment was discontinued for patients who developed erlotinib-related interstitial lung disease or grade 4 toxicities.

### Tissue correlative studies

Core or fine-needle aspiration (FNA) biopsy samples were used for the correlative studies. In cases where pretreatment core biopsy specimens were available, the biopsied tissue was analyzed for expression of Kirsten rat sarcoma viral oncogene homolog (K-ras); PTEN; total and phosphorylated Erk, Akt and mTOR, pS6K and 4E-binding protein 1 (P4EBP-1) by immunohistochemistry. Immunofluorescence staining and Laser Scanning Cytometry (LSC) were used to quantify the levels of expression of the above proteins when only FNA samples were available [10,11]. The LSC system (CompuCyte Corporation, Cambridge, MA) is an automated microscopy platform that enables the measurement of protein biomarkers at the resolution of individual cells. The system uses multiple lasers to measure levels for a panel of proteins (based on conjugated antibody fluorescence) and provides microscopy for the evaluation of cell morphology. The image analysis software automatically enumerates cells based on antibody selection criteria and reports a sensitive and quantitative measure of protein levels, termed "mean fluorescence intensity" (MFI). Prior to LSC analysis, the presence of tumor tissue was confirmed by light microscopy, and those areas containing tumor were selected for data acquisition on the LSC system. Each slide was placed on the motorized microscope stage and the selected regions were exposed to the scanning laser.

For quantitative measurement of the protein markers, the contour threshold was set to maximize the contouring of single cells.

To measure the protein markers in this study, we incubated formalin-fixed tissue specimens with rabbit anti-pS6 antibody (4857, CellSignaling, 1:1000), rabbit anti-p4EBP-1 antibody (2855, CellSignaling, 1:50), rabbit anti-PTEN antibody (9559, CellSignaling, 1:100), rabbit anti-pAKT antibody (124001, Calbiochem, 1:100), mouse anti-AKT antibody (2966, CellSignaling, 1:50), mouse anti-pERK antibody (M8159, Sigma, 1:100), rabbit anti-ERK antibody (442675, Calbiochem, 1:500), rabbit anti-pmTOR antibody (2976, CellSignaling, 1:50) or rabbit anti-mTOR antibody (SC-1549, Santa Cruz, 1:50). After incubation with these antibodies, the specimens were incubated with the appropriate secondary immunoglobulin G (IgG)-Cy5 conjugate antibody (Jackson ImmunoResearch Laboratories, West Grove, PA). Slides were scanned using a 200× objective and cell nuclei were contoured on the basis of the nuclear staining. The relative fluorescence levels for each antigen were plotted on a scattergram. The phosphorylated/total ratio of MFIs for the protein markers was computed.

### Statistics

The primary outcome of interest was 6-month survival. Thall and Simon's method was employed to perform continuous interim monitoring for efficacy [12]. Phase II data indicate that patients with gemcitabine-refractory, advanced pancreatic cancer have a 6-month survival rate of 29% and a median survival of 3.4 months [13]. This figure (29% 6-month survival) was referenced to compute the stopping rule. This prevented a high probability of stopping if the true success rate was ≥ 20%. The study would have been stopped at any time if we had determined there was a less than 1% chance that the surviving proportion in the experimental arm of the study was less than that from the historical control group. We assumed a uniform prior distribution for the surviving proportion for the new treatment and this distribution was updated as the study accrued patients. Using this rule, we would have stopped the study if the surviving proportion was not greater than 0/10, 1/17, 2/23, 4/33 or 5/38. Computer simulations were run to determine the operating characteristics of this rule. The probability of early stopping was 99% for a true surviving proportion of 0.05, 88% for a proportion of 0.1, 31% for a proportion of 0.2 and 6% for a proportion of 0.3. The expression of biomarkers before treatment was correlated with clinical parameters using the Cox proportional hazard model. Given the sample size, these analyses were exploratory.

## Results

Study A enrolled patients from December 2003 through January 2004. Study B enrolled patients from March 2008 through June 2008. All patients had experienced tumor progression while receiving gemcitabine prior to enrollment. Table 1 provides patient characteristics.

**Table 1:** Patient Characteristics (Study A and B)

Characteristic	Number
Age	63 (range 34-75)

ECOG PS 0*	3

ECOG PS 1	16

Males	12

Females	7

White	18

Hispanic	1

Prior surgery	6

Prior radiation	2

Prior chemotherapy regimens	

1	5

2	8

≥ 3	2

* ECOG PS: Eastern Co-operative Oncology Group Performance Status

Study A enrolled 5 patients; 4 actually received the planned therapy. The study was closed to accrual due to significant adverse effects (SAEs) resulting from study treatment. One patient in this study died within 1 month from rapid disease progression. One died within 1 month following a cerebrovascular accident; treatment response could not be evaluated. Two patients were withdrawn from the study because of SAEs and disease progression. The median number of treatment cycles was 0.75 (21 days), median OS was 44 days and PFS was 19 days. No responses occurred. Because of these events, the principal investigator (HX) recommended stopping the study; enrollment was stopped and the study closed to accrual.

Study B enrolled 16 patients; 15 received the planned therapy. The median number of cycles administered was 2 (56 days). PFS was 49 days and the OS was 87 days. No radiologic responses occurred. After reviewing the survival data, we concluded that the pre-specified median OS of 6 months was not likely to be reached and stopped the study for futility. Tables 2A and 2B depict study-related toxicities. As shown, systemic toxicities including fatigue, dehydration, and hyponatremia occurred with the combination. Lipid profile abnormalities were uncommon and below grade 2 in severity. Grade 2 skin rash occurred in 2 patients and was controlled with topical antibiotics and skin moisturizers.

**Table 2:** Study A; Number of Patients with Treatment-Related Toxicities and Study B; Number of Patients with Treatment-Related Toxicities

A		
Toxicities	Grade 3 or 4	Grade 5

Study A		

Hemorrhagic stroke	-	1

Pain	2	-

Fatigue	1	-

Constipation	1	-

Anorexia	1	-

Deep vein thrombosis	1	-

B		

Study B	Grade 2	Grade 3 or 4

Neutropenia	2	-

Hyponatremia	-	1

Diarrhea	-	1

Fatigue	-	1

Hyperglycemia	-	1

Mucositis	2	-

Pneumonia	2	-

Dehydration	2	-

Nausea	2	-

Rash	2	-

### Correlative data

**Figure 1:**
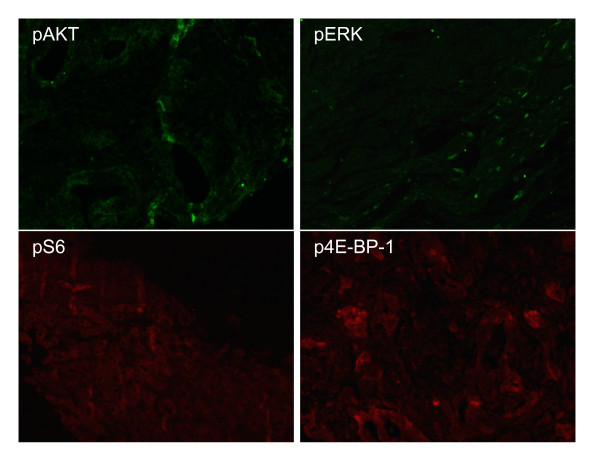
**Protein Expression by Immunofluorescence**. Pretreatment biopsies revealed increased pAkt/Akt ratio in tumor specimens as compared with non malignant pancreatic tissue. No such trends were noted for pErk/Erk or pmTOR/mTOR. Immunofluorescence results depicted above.

**Figure 2:**
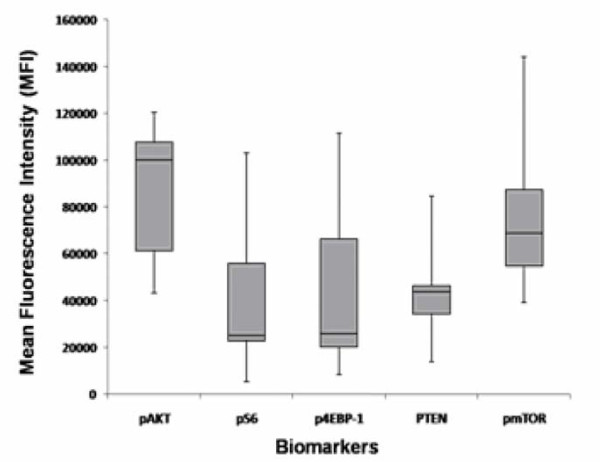
**Mean fluorescence intensity (MFI) of the biomarkers by laser scanning cytometry (LSC)**. For each biomarker, the box represents the middle half of the distribution of the data points stretching from the 25^th ^to the 75^th ^percentile. The line across the box represents the median. The lengths of the lines above and below the box are defined by the maximum and minimum data point values, respectively.

**Figure 3:**
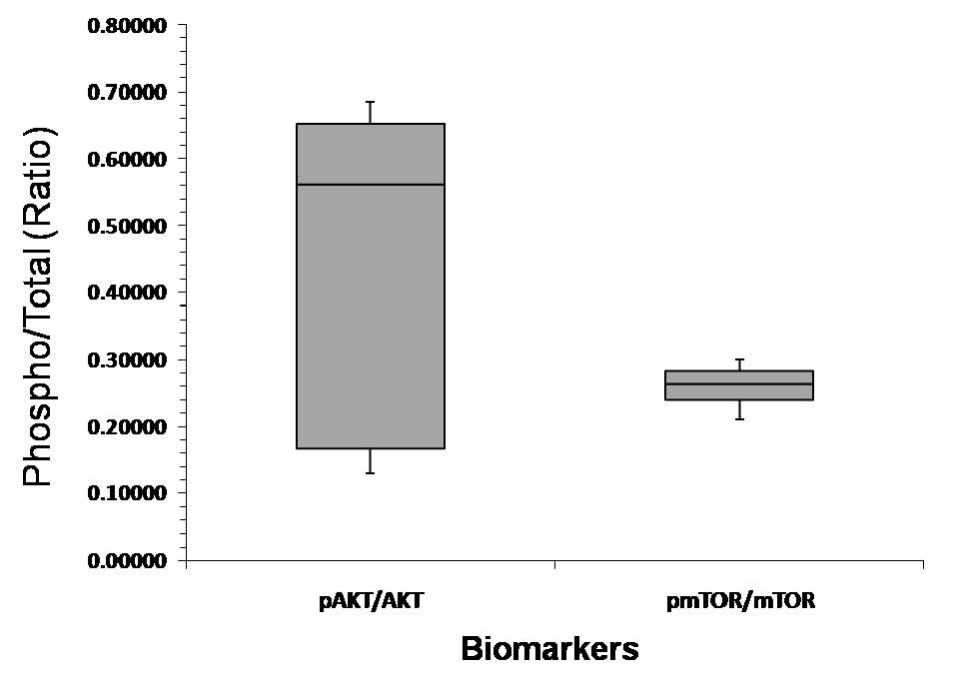
**Ratio of phosphorylated/total Akt and phosphorylated/total mTOR**. For each set of values, the box represents the middle half of the distribution of the data points stretching from the 25^th ^to the 75^th ^percentile. The line across the box represents the median. The lengths of the lines above and below the box are defined by the maximum and minimum data point values, respectively.

Figures 1, 2 and 3 show the MFIs of the biomarkers acquired by LSC as represented in box plots. Activation of Akt, as assessed by the pAkt/Akt ratio, was noted. No significant trends were noted in the other biomarkers studied.

## Discussion

The mTOR is a validated target in many human cancers, and rapamycin analogs (rapalogs) have shown promising results in various tumor types, including non-Hodgkin's lymphoma, sarcoma, glioblastoma and endometrial cancer. The PI3K/Akt/mTOR pathway is activated in pancreatic cancer by overexpression or activation of EGFR and insulin-like growth factor (IgF1R), by PTEN loss or secondary to k-ras mutation and activation of the Ras/Raf/MEK pathway. The investigation of rapalogs for pancreatic cancer treatment is therefore based on sound rationale.

We observed dose-limiting toxicity from standard doses of temsirolimus in Study A and a lack of antitumor activity in both studies (A and B). The reason for the toxicity noted with standard doses of temsirolimus or everolimus is not clear. Patients with gemcitabine-refractory pancreatic cancer can be frail and are often not the best candidates for aggressive systemic therapy. However, our institutional experience using capecitabine and oxaliplatin in this patient group reveals that patients with gemcitabine-refractory pancreatic cancer and good performance status can tolerate and derive benefit from systemic chemotherapy [2]. Therefore, the high toxicity particularly observed with temsirolimus was surprising, despite the use of an approved dose. The concern was raised that the "toxicity" noted resulted at least in part from disease progression. While this degree of toxicity did not occur in Study B, the study was discontinued after enrolling 15 patients, because of disease progression in all patients.

The weekly scheduling of everolimus was based on the dosing schedule identified in our phase I study. Taberbero et al. suggest that daily dosing of everolimus may result in a more sustained inhibition of S6 kinase than intermittent dosing [14]. However, Wolpin et al, recently reported no clinically relevant anti-tumor effect in a recent phase II trial of daily everolimus for patients with advanced, gemcitabine-refractory pancreatic cancer [15]. Therefore, we do not believe that the schedule of everolimus used in Study B resulted in the absence of antitumor effect.

Our recent studies revealed that mTOR inhibitors such as rapamycin and temsirolimus increase Akt phosphorylation/activation and cyclin D1 expression levels in pancreatic cancer cells [16]. Similar results were reported by Wan et al., who suggested that mTOR inhibitors might eventually promote cellular proliferation and survival if the Akt activation goes unchecked [17]. In support of this possibility, pancreatic cancer cells were observed by Wan et al to become more resistant to the mTOR inhibitors beyond 72-96 hours of continuous treatment *in vitro*. Li et al, also observed that the phosphorylation of IRS-1 on Ser612, a modification that likely inhibits IRS-PI3K interactions, was abolished by rapamycin and CCI-779. It is therefore conceivable that IRS-Ser612 is a mTOR-regulated site that is part of a negative feedback loop, and that mTOR inhibitors enhance pAkt levels in pancreatic cancer cells by blocking its phosphorylation [18]. Why this feedback loop is more relevant in pancreatic cancer than in other cancer types is unclear at this time. Whether the paradoxical Akt activation secondary to mTOR inhibition led to the increased toxicity or rapid tumor progression in Study A cannot be stated with any degree of certainty as no post-treatment biopsies were performed.

The sensitivity of cancer cell lines to erlotinib depends at least partially on the inhibition of the PI3K/Akt/mTOR pathway. The erlotinib-rapamycin combination was found to have a synergistic cytocidal effect and erlotinib alone could inhibit rapamycin-induced Akt activation in non-small cell lung cancer cell lines [19]. These data supported the investigation of the erlotinib-everolimus combination in Study B. However, in Study B, we observed neither clinically significant antitumor activity nor disease stability. One possible explanation may be that most pancreatic cancers are k-ras mutated, which may induce baseline Akt activation in pancreatic cancer. Indeed our pretreatment biopsies revealed an elevated pAkt/Akt ratio in pancreatic cancer cells pre-treatment. It must be noted however, that both studies did not include post-treatment biopsies; therefore, we cannot definitively rule out an erlotinib effect on Akt activation. One can hypothesize, based on our results, that Akt inhibition as a therapeutic strategy for pancreatic cancer should include the simultaneous inhibition at several points along the PI3K pathway. Ongoing studies are investigating mTOR inhibitors in combination with IgF1-R or direct Akt antagonists.

Standard second-line chemotherapy regimens for advanced pancreatic cancer at the present time include oxaliplatin with fluoropyrimidines or capecitabine [2]. Clinical trials that are based on molecular profiling, or which include one of the above standard regimens should be encouraged.

## Conclusions

PI3K inhibition may be an important therapeutic strategy for pancreatic cancer. However, we did not observe a clinically relevant anti-tumor effect with the mTOR inhibitor temsirolimus or with the everolimus + erlotinib combination.

## Competing interests

Novartis for research support; OSI Pharmaceuticals for the supply of erlotinib.

## Authors' contributions

MJ directed the overall study design and accrual, RJ participated in study design and accrual, HX directed the study design of the temsirolimus trial, GV participated in study enrollment and manuscript writing, SR participated in the correlative studies, DR participated in correlative studies, YZ participated in the pathology study, RAW participated in the study accrual and manuscript writing, JA participated in manuscript writing and study accrual. All authors have read and approved the manuscript.

## Authors' information

These studies were conducted by the authors at the MD Anderson Cancer Center, Houston, TX, USA.

## Pre-publication history

The pre-publication history for this paper can be accessed here:

http://www.biomedcentral.com/1471-2407/10/368/prepub
